# In Vivo Wound Healing Activity of* Abrus cantoniensis* Extract

**DOI:** 10.1155/2016/6568528

**Published:** 2016-12-29

**Authors:** Qi Zeng, Hui Xie, Hongjin Song, Fayu Nie, Jiahua Wang, Dan Chen, Fu Wang

**Affiliations:** School of Life Science and Technology, Xidian University, Xi'an, China

## Abstract

*Abrus cantoniensis* (Leguminosae sp.) is a traditionally used remedy for treating rheumatism, blood stasis, and internal injuries. In order to reveal a new insight of the utilization of the plant, solvent extraction by ethyl acetate (EA) was performed in order to evaluate the plant extracts' in vivo excision and incision-wound potentials with models. The contents of the EA fraction, wound healing activity, acute oral toxicity, and acute dermal toxicity were studied. As a result, the main chemical constituents of the EA fraction were alkaloids, flavonoids, and steroids. The acute oral toxicity test results and assessment of skin hypoallergenicity showed that the plant extract was safe at LD50 as high as 5000 mg/kg. Both excision and incision model tests results indicated that the EA fraction of* A. cantoniensis* showed a significant wound healing capacity at a concentration of 5% (v/w) (*p* < 0.01) as observed by the increased wound contraction, decreased epithelialization time, and increased hydroxyproline content compared to the ones of the controls. The present study showed that the EA fraction of* A. cantoniensis* possesses potential wound healing activities and provided recent results for the use of* A. cantoniensis* for wound curing.

## 1. Introduction

Wound healing is often considered as a major problem in clinical practice. It is a complex process occurring after injury and is as old as mankind [1]. Wound healing is accomplished by inflammation, proliferation, differentiation, migration, organization, and remodeling of cells inside and around the vicinity of the injury [2]. The proliferative phase consists of angiogenesis, collagen deposition, epithelialization, and wound contraction. The aim of treating wounds is to shorten the time of healing and reduce the risks of undesired complications [3]. Over three-quarters of the world population relies mainly on plants and plant extracts for health care [4]. And for the remedy of wound healing, more than 400 species of plants are identified as potentially useful alternative medicine [5].

Traditional Chinese medicine “Jigucao” belongs to the* Abrus* genus with its Latin name as* Abrus cantoniensis* Hance.* A. cantoniensis* is found in the Hunan, Guangdong, and Guangxi provinces [6]. In China, it is traditionally used against ailments like acute and chronic hepatitis, cirrhosis, cholecystitis, stomach pain, rheumatism, blood stasis, and internal injuries [7, 8]. Previous studies showed that this plant contained various chemical constituents including triterpenoids, steroids, flavonoids, anthraquinones, phenolic acids, and alkaloids [9–17]. Indeed, the medicinal activities of the plant are mainly due to the presence of the constitutive secondary metabolites. However, no study has evaluated the wound healing activity of* A. cantoniensis*. In this context, the aim of the present study was to investigate the wound healing activity of the ethyl acetate fraction of* A. cantoniensis *using the Swiss Albino mice as host for the wound models.

## 2. Materials and Methods

### 2.1. Plant Material

Whole plants of* A. cantoniensis* were collected from Meizhou, Guangdong, China, in July 2012, and identified by the pharmacist Yan-rong Li from the Xijiao Hospital of the Meijiang District, Meizhou, China. A voucher specimen (JGC201207) was deposited at the School of Life Science and Technology, Xidian University, Shaanxi, China.

### 2.2. Animals

Swiss Albino mice (18–22 g, 8–10 weeks of age) were obtained from the animal house of the Xi'an Jiaotong University Health Science Center, China. The mice were housed at room temperature (25 ± 1°C) and were subjected to a 12 h light/12 h dark cycle. All the experiments were conducted in accordance with the internationally accepted laboratory animal use and care guidelines [18] and the protocol was approved by the School of Pharmacy's Ethics Committee. Mice were acclimatized for one week before the study, and during the experiment the mice were housed individually in their cages so as to avoid biting and possible wound scratch among each other. The mice were provided with water and food pellets before and until the end of the experimental period. The animal study procedures were approved and followed by the Xian Jiaotong University Animal Care and Use Committee (number XJTULAC2016-412).

### 2.3. Extraction and Fractionation

The air-dried and powdered whole plants of* A. cantoniensis* (5 kg) were extracted with 95% ethanol six times (20 L × 24 h) at room temperature. After evaporation under vacuum, the residue (167.6 g) was then suspended in water and partitioned with petroleum ether (PE), EA, and* n*-butanol (BuOH), respectively. Thus, the extracts from the three different solvents were obtained, submitted separately to solvent evaporation, and named consequently as petroleum ether extract fraction (PE fraction, 42.1 g), ethyl acetate extract fraction (EA fraction, 27.6 g), and n-butanol extract fraction (BuOH fraction, 60.2 g).

### 2.4. Formulation of the EA Fraction

Two types of formulations (suspension and simple ointment) were prepared. The suspension was prepared following the procedure described by the Encyclopedia of Pharmaceutics Excipient [19] with modifications for the EA fraction. Namely, 2.5 g of the EA fraction was dissolved in 0.75~1.5 mL Tween 80 and grinded until obtaining a white nonhomogenous mixture. Then 100 *μ*L of water was added and the mixture was grinded. The addition of water and grinding step were repeated until the EA fraction was fully dissolved. Finally the volume of the mixture was adjusted to a constant volume of 15 mL. The simple ointment was prepared based on the procedure of Pharmaceutics [20] while using the reduced formula as the base for the EA fraction. More precisely, 5 g of sodium carboxymethylcellulose (Na-CMCC) was mixed in water at 70°C with continuous stirring during 3 hours until no solid particles were found in the mixture. The mixture was then removed from the heating bath and cooled under stirring to obtain the ointment base. In order to obtain the control ointment, 30 mg of nitrofurazone was added to the ointment base resulting in 0.2% (w/v) nitrofurazone ointment. The EA fraction containing ointment was prepared with 10 mL of Na-CMCC in order to form, respectively, 5% and 10% (w/v) EA fraction ointments. All the simple ointments were stored at 4°C.

### 2.5. Preliminary Phytochemical Analysis

The EA fraction was screened for the presence of secondary metabolites, including alkaloids, saponins, flavonoids, tannins, steroids, and terpenoids by following the procedures described elsewhere [21].

#### 2.5.1. Alkaloids

The test for alkaloids was carried out on 10 mg of EA fraction mixed with 5 mL of ammoniacal chloroform and 2.5 mL of chloroform. After filtration, the supernatant was shaken with drops of 0.5 M sulfuric acid. The appearance of a creamy precipitate indicated the presence of alkaloids.

#### 2.5.2. Saponins

The test for saponins was carried out by adding 10 mg of EA fraction shaken vigorously with 1 mL of ethyl ether and 3 mL of a 2 N solution of hydrochloride (HCl). The appearance of a precipitate indicated the presence of saponins.

#### 2.5.3. Flavonoids

A sample containing 5 mg of the EA fraction was dissolved in 5 mL of absolute ethanol and treated with a few drops of concentrated HCl and 0.2 g of magnesium ribbon. The appearance of a pink-red color indicated the presence of flavanoids.

#### 2.5.4. Tannins

A sample prepared with 5 mg of EA fraction was dissolved in 10 mL of 70% ethanol. The sample was then diluted with sterile distilled water at a ratio of 1 : 2 (v/v). Three drops of 10% (w/v) ferric chloride solution was then added. The appearance of a blue to black precipitate indicated the presence of tannins.

#### 2.5.5. Steroids and Terpenoids

Steroids and terpenoids were detected using the Liebermann-Burchard reaction. A solution containing 5 mg of EA fraction dissolved in chloroform was filtered. The filtrate (2 mL) was added to 2 mL of acetic anhydride and 50% concentrated sulfuric acid. A blue-green ring indicated the presence of steroids while a red color indicated the presence of terpenoids.

### 2.6. Acute Toxicity Studies

#### 2.6.1. Acute Oral Toxicity Study

Fifteen Swiss Albino mice of both sex weighing between 18 g and 22 g (average weight of 20 g) were used for the acute oral toxicity study. A suspension of the EA fraction was administered following the OECD guideline number 420, starting at a dose level of 2000 mg/kg up to 5000 mg/kg (0, 2000, 3000, 4000, and 5000 mg/kg; five groups of three mice) [22]. Before administration of the EA fraction suspension, all the mice were physically active and consumed food and water regularly. The mice were treated with a suspension of the EA fraction by intragastric administration (0.5 mL) and observed for signs of acute toxicity within 48 h. The mice health was further monitored during the following 7 days in order to detect any general signs of subacute toxicity.

#### 2.6.2. Acute Dermal Toxicity Study

Fifteen mice were used for the skin irritation test. Mice showing normal skin texture were housed individually in a cage. According to the Meeh-Rubner formula, the body surface area of the mice was calculated. Mice were shaved at the dorsal area of the trunk that represented 10% of the skin area 24 h before the study. The mice received a dose of 2000 mg/kg of EA fraction by applying the ointment over the shaved area. The mice were observed for an adverse skin reaction at grading interval of 1, 4, 12, and 24 h.

### 2.7. Grouping and Dosing of Animals

Mice were divided into four groups: a negative and positive control groups and two test groups. Six mice were used in each group. Mice of the negative control group (group A) were treated with the simple ointment base. Groups B and C were treated with 5% (w/w) and 10% (w/w) of the EA fraction, respectively. The mice in group D were treated with 0.2% (w/v) of nitrofurazone as positive control.

### 2.8. Wound Healing Studies

#### 2.8.1. Excision Wound Model

Mice were anesthetized by subcutaneous injection of chloral hydrate (1 mL/kg) and 1% atropine. The back of the mice was further shaved. A 215 mm^2^ (representing 3.2% of the weight of mice) circular area was marked and the surface of the marked area was carefully excised by using sharp sterilized scissors [23]. After 24 h of wound creation, the ointments were gently applied to cover the wounded area once per day until reaching complete healing [24]. Wound area, wound contraction, epithelialization period, and hydroxyproline content were monitored during the whole healing process.

#### 2.8.2. Wound Area and Wound Contraction

The wound was monitored and the healing area was calculated by using semitransparent tracing paper. The tracing paper was placed on a 1 mm^2^ graph sheet and traced out. The area was measured daily and the percentage wound closure was calculated by the following formula:

(1) %  Wound  contraction=Wound  area  on  day  0−Wound  area  on  day  nWound  area  on  day  0×100.

#### 2.8.3. Estimation of the Hydroxyproline Content

**Figure 1:**
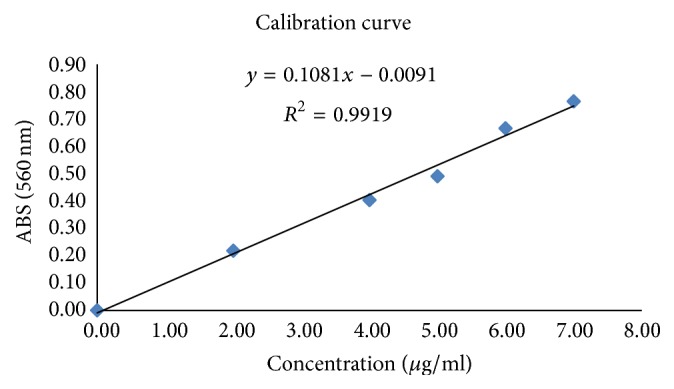
Calibration curve of hydroxyproline specifically plotted for the EA fraction. The standard hydroxyproline solutions had the following concentrations: 7, 6, 5, 4, 2, and 0 *μ*g/mL. The hydroxyproline content was determined by the method described by Leach [25] and using a UV-detection at 560 nm on a spectrophotometer.

A calibration curve was plotted using standard hydroxyproline solutions in order to determine the hydroxyproline content of the tissue. The hydroxyproline content was determined by following the method described by Leach [25]. The calibration curve of the hydroxyproline content in the EA fraction is depicted in Figure 1. The injured mice were subjected to the ointment formulation treatment during 10 days and then sacrificed on the 11th day using a high dose of diethyl ether. The healed tissue was excised and the water was absorbed with filter paper, before grinding the tissue. A sample weighing 0.15 g of wound tissue was then hydrolyzed with 3 mL of 6 N hydrochloric acid for 24 h at 110°C in a sealed conical glass flask. The pH of the hydrolysate was neutralized to pH 8.0 ± 0.2 [26]. The supernatant solution (1 mL) was removed delicately with a pipette from each of the hydrolysates and submitted to the standard treatment. The hydroxyproline content of the samples was determined based on the equation extracted from the calibration curve.

#### 2.8.4. Incision Wound Model

Anesthetized mice were shaved and a straight line was marked at a distance of 1 cm from the paravertebral ganglia. An incision wound of 3 cm long and of full skin thickness, parallel to the paravertebral region, was made with a sterile scalpel [27]. The wound was then closed by interrupted sutures having 1 cm intervals. The mice were treated with ointments 24 h after the incision and during nine days. The sutures were removed on the 8th day. The tensile strength of the wound was measured on the 10th day [24].

#### 2.8.5. Measurement of the Tensile Strength

The breaking strength of the wound on each animal was measured by using the constant water flow method [28].

### 2.9. Statistical Analysis

The statistical differences were evaluated using the software IBM SPSS Statistics v. 19.0.0 (New York, USA). The normality of the distributions was evaluated through the Kolmogorov-Smirnov's test and the differences were evaluated using the software One-Way ANOVA associated with Scheffe's test (for normal distributions) or the nonparametric Mann–Whitney test for the rest. Differences were considered significant when *p* < 0.05.

## 3. Results

### 3.1. Phytochemical Screening

Analyses of the EA fraction of* A. cantoniensis* indicated the presence of alkaloids, flavonoids, and steroids (Table 1) as consistent with former reports [17]. However, our EA fraction did not contain saponins and terpenoids in contrast to previous studies [9–15]. Indeed, the polarity of EA fraction hinders the retention of saponins and terpenoids molecules.

### 3.2. Acute Toxicity Studies

During the 7 days of the acute oral toxicity observation period, none of the mice died nor showed any adverse reaction such as towering hair, exophthalmos, muscle paralysis, convulsions, breathing difficulties, teeter, coma, or incontinence. The LD_50_ of the EA fraction was greater than 5000 mg/kg. Concerning the skin irritation test, the swelling and erythema did not appear on the test group during the whole experimental period (14 days). Therefore, we estimate a safe dosage of EA fraction superior to 2000 mg/kg.

### 3.3. Excision Wound Study

**Figure 2:**
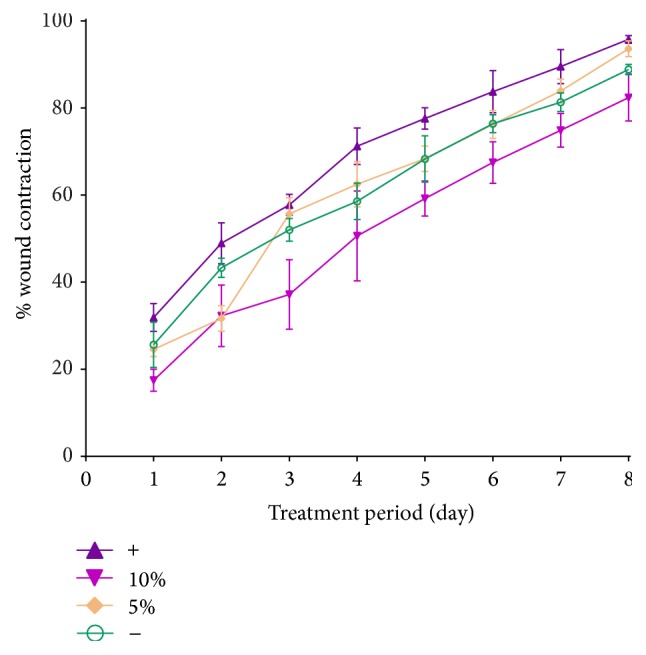
Effect of the EA fraction formulated in ointments on the percentage of wound contraction. The excision wound models were treated with 5% (w/v) and 10% (w/v) of the EA fraction. Negative control group and the blank group were treated with 0.2% (w/v) nitrofurazone and simple ointment.

**Figure 3:**
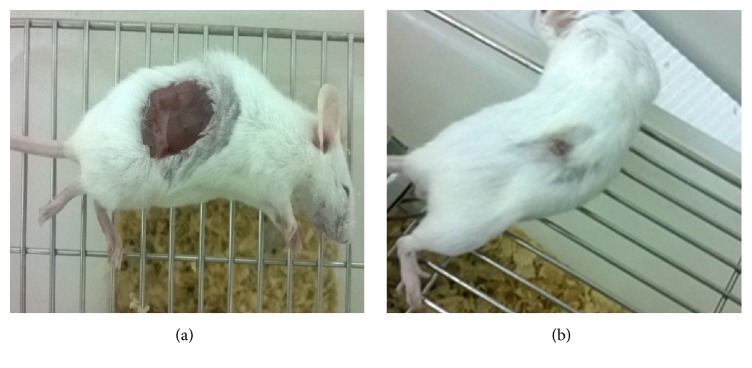
Photograph of appearance of a healed excision wound: (a) day 0 and (b) day 10.

The ointment containing 5% (w/v) of the EA fraction showed an increased wound contraction rate compared to the one of the negative control group (e.g., on the 8th day, we observed a wound healing of 94.48% for mice treated with 5% EA against 84.92% for the control group) (Table 2). From the 6th day, the contraction value was even slightly higher for the 5% EA fraction treated mice than for the mice treated with the reference drug nitrofurazone. However, a higher dose of EA fraction (10%, (w/v)) did not show any significant difference compared to the results obtained by the negative control group. Moreover, extrapolation of the results as represented by the trend lines in Figure 2, supports the positive effect of EA fraction in would healing. The excision wound was healed on the 10th day for the mice treated with 5% (w/v) ointment (Figure 3). As showed in Table 3, the hydroxyproline content of the 5% EA fraction was 6.264 *μ*g/g while the one of the reference drug group was 7.448 *μ*g/g. These values are nearly the double of the value detected for the control group (3.766 *μ*g/g). Conversely, the hydroxyproline content of the 10% EA fraction exhibited a lower value as in the case of the control group.

### 3.4. Tensile Strength of the Incision Wound

The values of the tensile strength of the incision wound treated with the EA fraction on day 10 are presented in Table 4. Both the high dose (10%, (w/v)) and the low dose (5%, (w/v)) of EA fraction exhibited wound breaking strength values comparable to the one of the control group. The tensile strength of the mice treated with 5% (w/v) EA fraction ointment was higher than the one of the group treated with 10% (w/v) The best healing was observed for the group treated with the EA fraction ointment on the 10th day of treatment.

## 4. Discussion

Wounds are physical injuries of the skin. Healing is a complex process initiated in response to an injury and restores the function and integrity of damaged tissues [3]. Unpublished results showed that the EA fraction showed the highest antibacterial activity among the four other solvent extracts of* A. cantoniensis *(PE, EA, BuOH, and EtOH). Therefore, the aim of this work was to evaluate the wound healing activity of the EA fraction. The results of this study showed, for the first time, the enhancement of the wound contraction rate and reduction of the healing time in mice treated with an ointment containing the EA fraction of* A. cantoniensis*. This result highlights a new possible usage of the traditional medicine plant.

The ointment formulation of the medicinal plant could achieve wound care healing [29]. Therefore, the EA fraction was prepared as an ointment formulation in the excision and incision wound experiments. Moreover, the ointment of the EA fraction showed low allergenicity to the skin. The suspension formulation of the EA fraction was used for the acute toxicity test. An acceptable safety high dosage of 5000 mg/kg bw was determined. These results indicated the safe usage of the EA fraction of* A. cantoniensis* on mice and revealed a potential application for clinical issues.

The lower dose (5%, (w/v)) of the EA fraction accelerated the wound healing (*p* < 0.01) when compared to the results obtained for the negative control groups for both excision and incision tests. Specifically, the 5% EA fraction ointment exhibited a better healing effect than the reference drug (nitrofurazone) in the excision tests. The whole healing period was shortened to ten days when the mice were treated with the ointment containing 5% of EA fraction. Hydroxyproline is a major amino-acid constituent of collagen and serves as marker for collagen content in tissue samples [30]. An increase in the hydroxyproline content indicates an increase in the collagen synthesis, which in turn leads to improved wound healing activity. In this study, the hydroxylproline content of the tissues from mice treated with 5% EA fraction ointment was significantly higher than the ones detected for the negative control mice. This result demonstrated the efficient wound healing activity of a low dose of EA fraction in the excision model. However, a higher dosage of 10% EA fraction in the ointment was not beneficial as shown by the results obtained in both test models and the hydroxyproline content determination. In our previous antibacterial study, the efficient dosage range of EA fraction exhibited a similar trend of this result. In parallel, it could be deduced that the wound healing activity of the EA fraction might be also related to its antimicrobial activity that occurs at a specific dosage interval.

The antimicrobial and wound healing activities might be due to the presence of alkaloids, flavonoids, and steroids in the preliminary phytochemical analysis (Table 1). Other medicinal plants, which were identified as potentially useful for wound healing, contained similar constituents. These metabolites were suggested to play a critical role in the wound healing process by increasing the rate of wound contraction, epithelialization, and prevention of secondary bacterial infections that would have complicated and delayed the wound healing [31–34]. The absence of saponins and terpenoids in the EA fraction might indicate that these compounds do not have affinity with the polar ethyl acetate extraction solvent. As a result, further phytochemical studies should be investigated to pinpoint the active compounds of* A. cantoniensis*.

*A. cantoniensis* is a basic plant of the traditional medicine “Jigucao.” It appears in the daily life as the Guangdong herbal tea or as an ingredient in soups. The ancient and common usage of this plant demonstrated its safe character and indicated the potential development in the medicinal field. With the rising respect towards “medicine and food homology,” we believe that* A. cantoniensis* has a high potential medicinal value. Yet, the substantial constituents of the wound healing activity and action mechanisms were not determined. The next study should be focused on the phytochemistry detection, healing markers analysis, and in vivo dose-effect relationship.

## 5. Conclusion

The present study revealed the wound healing activity of the EA fraction of* A. cantoniensis*. Wound contraction, increased breaking strength of the repaired tissue, and the increased hydroxylproline content support the observed wound healing. As a conclusion, the results of the study indicated a new view of this medicinal plant for the usage of wounds curing.

## Figures and Tables

**Table 1:** Qualitative analysis of bioactive compounds in the EA fraction of *A. cantoniensis*.

	Alkaloids	Saponin	Flavanoids	Tannins	Steroids	Terpenoids
EA fraction	+	−	+	−	+	−

Note: present (+); absent (−).

**Table 2:** Effect of the EA fraction formulated in ointment on the percentage of wound contraction.

Group (*n* = 6)	Treatment period (day)			
	2nd	4th	6th	8th
0.2 w/v nitrofurazone	58.46 ± 12.05^*∗*^	74.62 ± 7.15^*∗∗*^	82.23 ± 7.25^*∗*^	92.73 ± 4.28^*∗*^
10% EA fraction	47.38 ± 19.21	57.85 ± 14.85	71.08 ± 8.21	85.54 ± 7.00
5% EA fraction	53.35 ± 8.12^*∗*^	66.14 ± 9.41^*∗*^	83.93 ± 9.16^*∗*^	94.48 ± 3.04^*∗∗*^
Simple ointment	44.04 ± 7.63	56.96 ± 5.58	70.71 ± 8.20	84.92 ± 5.84

Note: ^*∗*^0.01 < *p* < 0.05; ^*∗∗*^*p* < 0.01.

**Table 3:** Effect of the EA fraction on the hydroxyproline content of the granulation tissue of the excision wound.

Group (*n* = 6)	Hydroxyproline content (*µ*g/g)
0.2 w/v nitrofurazone	7.448 ± 1.442^*∗∗*^
10% EA fraction	3.562 ± 1.109
5% EA fraction	6.264 ± 1.617^*∗∗*^
Simple ointment	3.766 ± 0.875

Note: ^*∗∗*^*p* < 0.01.

**Table 4:** Effect of EA fraction on the tensile strength.

Group	Tensile strength (g) (% tensile strength)
0.2 w/v nitrofurazone	242.83 ± 59.82^*∗∗*^
10% EA fraction	171.17 ± 23.30^*∗*^
5% EA fraction	197.83 ± 46.01^*∗∗*^
Simple ointment	134.50 ± 26.37

Note: ^*∗*^0.01 < *p* < 0.05; ^*∗∗*^*p* < 0.01.

## References

[1] Kokane D. D., More R. Y., Kale M. B., Nehete M. N., Mehendale P. C., Gadgoli C. H. (2009). Evaluation of wound healing activity of root of *Mimosa pudica*. *Journal of Ethnopharmacology*.

[2] Mutsaers S. E., Bishop J. E., McGrouther G., Laurent G. J. (1997). Mechanisms of tissue repair: from wound healing to fibrosis. *International Journal of Biochemistry and Cell Biology*.

[3] MacKay D., Miller A. L. (2003). Nutritional support for wound healing. *Alternative Medicine Review*.

[4] Yogisha S., Raveesha K. A. (2009). *In vitro* antibacterial effect of selected medicinal plant extracts. *Journal of Natural Products*.

[5] Ghosh P. K., Gaba A. (2013). Phyto-extracts in wound healing. *Journal of Pharmacy and Pharmaceutical Sciences*.

[6] Wei Z. (1994). *Flora of China*.

[7] Song L. R. (1999). *Chinese Materia Medica*.

[8] Yan Y. Q., Yu C. L., Huang T. K. (1996). *Chinese Medicine Dictionary*.

[9] Takashi T., Shuichi H., Toshihiro N. (1989). New triterpenoid sapogenols from *Abrus cantoniensis* (I). *Chemical and Pharmaceutical Bulletin*.

[10] Miyao H., Sakai Y., Takeshita T., Kinjo J., Nohara T. (1996). Triterpene saponins from *Abrus cantoniensis* (Leguminosae). I. Isolation and characterization of four new saponins and a new sapogenol. *Chemical and Pharmaceutical Bulletin*.

[11] Miyao H., Sakai Y., Takeshita T., Ito Y., Kinjo J., Nohara T. (1996). Triterpene saponins from *Abrus cantoniensis* (Leguminosae). II. Characterization of six new saponins having a branched-chain sugar. *Chemical and Pharmaceutical Bulletin*.

[12] Sakai Y., Takeshita T., Kinjo J., Ito Y., Nohara T. (1990). Two new triterpenoid sapogenols and a new saponin from Abrus cantoniensis (II). *Chemical and Pharmaceutical Bulletin*.

[13] Chiang T. C., Chang H. M. (1982). Isolation and structural elucidation of some sapogenols from *Abrus cantoniensis*. *Planta Medica*.

[14] Mak T. C. W., Chiang T.-C., Chang H.-M. (1982). X-ray crystal structures of cantoniensistriol and sophoradiol: two oleanane-type triterpenes from the roots of *Abrus cantoniensis* Hance. *Journal of the Chemical Society, Chemical Communications*.

[15] Miyao H., Arao T., Udayama M., Kinjo J., Nohara T. (1998). Kaikasaponin III and soyasaponin I, major triterpene saponins of *Abrus cantoniensis*, act on GOT and GPT: influence on transaminase elevation of rat liver cells concomitantly exposed to CCl_4_ for one hour. *Planta Medica*.

[16] Wong S. M., Chiang T. C., Chang H. M. (1982). Hydroxyanthraquinones from *Abrus cantoniensis*. *Planta Medica*.

[17] Shi H.-M., Wen J., Tu P.-F. (2006). Chemical constituents of *Abrus cantoniensis*. *Chinese Traditional and Herbal Drugs*.

[18] Institute for Laboratory Animal Research (ILAR) (1996). *Guide for the Care and Use of Laboratory Animals*.

[19] Luo M. S., Gao T. H. (2006). Encyclopedia of pharmaceutics excipient, Sichuan. *Sichuan Publishing House of Science & Technology*.

[20] Cui D. F. (2011). *Pharmaceutics*.

[21] Malahubban M., Alimon A. R., Sazili A. Q., Fakurazi S., Zakry F. A. (2013). Phytochemical analysis of *Andrographis paniculata* and *Orthosiphon stamineus* leaf extracts for their antibacterial and antioxidant potential. *Tropical Biomedicine*.

[22] The traditional Chinese medicine, natural medicine acute toxicity test technology guiding principle subject group, Chinese medicine, natural medicine acute toxicity research techniques guiding principle, 2005

[23] Akkol E. K., Koca U., Peşin I., Yilmazer D., Toker G., Yeşilada E. (2009). Exploring the wound healing activity of *Arnebia densiflora* (Nordm.) Ledeb. by in vivo models. *Journal of Ethnopharmacology*.

[24] Süntar I. P., Akkol E. K., Yilmazer D. (2010). Investigations on the in vivo wound healing potential of Hypericum perforatum L.. *Journal of Ethnopharmacology*.

[25] Leach A. (1960). *Notes on a Modification of the Neuman and Logan Method for the Determination of the Hydroxyproline*.

[26] Sanwal R., Chaudhary A. (2011). Wound healing and antimicrobial potential of *Carissa spinarum* Linn. in albino mice. *Journal of Ethnopharmacology*.

[27] Deshmukh P. T., Fernandes J., Atul A., Toppo E. (2009). Wound healing activity of Calotropis gigantea root bark in rats. *Journal of Ethnopharmacology*.

[28] Ilango K., Chitra V. (2010). Wound healing and anti-oxidant activities of the fruit pulp of Limonia acidissima linn (Rutaceae) in rats. *Tropical Journal of Pharmaceutical Research*.

[29] Odimegwu D. C., Ibezim E. C., Esimone C. O., Nworu C. S., Okoye F. B. C. (2008). Wound healing and antibacterial activities of the extract of Dissotis theifolia (Melastomataceae) stem formulated in a simple ointment base. *Journal of Medicinal Plants Research*.

[30] Lin Z.-Q., Kondo T., Ishida Y., Takayasu T., Mukaida N. (2003). Essential involvement of IL-6 in the skin wound-healing process as evidenced by delayed wound healing in IL-6-deficient mice. *Journal of Leukocyte Biology*.

[31] Mekonnen A., Sidamo T., Asres K., Engidawork E. (2013). In vivo wound healing activity and phytochemical screening of the crude extract and various fractions of Kalanchoe petitiana A. Rich (Crassulaceae) leaves in mice. *Journal of Ethnopharmacology*.

[32] Shailajan S., Menon S., Pednekar S., Singh A. (2011). Wound healing efficacy of *Jatyadi Taila*: *In vivo* evaluation in rat using excision wound model. *Journal of Ethnopharmacology*.

[33] Sasidharan S., Nilawatyi R., Xavier R., Latha L. Y., Amala R. (2010). Wound healing potential of Elaeis guineensis Jacq leaves in an infected albino rat model. *Molecules*.

[34] Reddy J. S., Rao P. R., Reddy M. S. (2002). Wound healing effects of *Heliotropium indicum*, *Plumbago zeylanicum* and *Acalypha indica* in rats. *Journal of Ethnopharmacology*.

